# pH-Sensitive Drug Delivery System Based on Chitin Nanowhiskers–Sodium Alginate Polyelectrolyte Complex

**DOI:** 10.3390/ma15175860

**Published:** 2022-08-25

**Authors:** Natallia V. Dubashynskaya, Valentina A. Petrova, Dmitry P. Romanov, Yury A. Skorik

**Affiliations:** 1 Institute of Macromolecular Compounds of the Russian Academy of Sciences, Bolshoy pr. V.O. 31, 199004 St. Petersburg, Russia; 2 Institute of Silicate Chemistry of the Russian Academy of Sciences, Adm. Makarova emb. 2, 199034 St. Petersburg, Russia

**Keywords:** chitin nanowhiskers, sodium alginate, metronidazole, vaginal delivery systems

## Abstract

Polyelectrolyte complexes (PECs), based on partially deacetylated chitin nanowhiskers (CNWs) and anionic polysaccharides, are characterized by their variability of properties (particle size, ζ-potential, and pH-sensitivity) depending on the preparation conditions, thereby allowing the development of polymeric nanoplatforms with a sustained release profile for active pharmaceutical substances. This study is focused on the development of hydrogels based on PECs of CNWs and sodium alginate (ALG) for potential vaginal administration that provide controlled pH-dependent antibiotic release in an acidic vaginal environment, as well as prolonged pharmacological action due to both the sustained drug release profile and the mucoadhesive properties of the polysaccharides. The desired hydrogels were formed as a result of both electrostatic interactions between CNWs and ALG (PEC formation), and the subsequent molecular entanglement of ALG chains, and the formation of additional hydrogen bonds. Metronidazole (MET) delivery systems with the desired properties were obtained at pH 5.5 and an CNW:ALG ratio of 1:2. The MET–CNW–ALG microparticles in the hydrogel composition had an apparent hydrodynamic diameter of approximately 1.7 µm and a ζ-potential of −43 mV. In vitro release studies showed a prolonged pH-sensitive drug release from the designed hydrogels; 37 and 67% of MET were released within 24 h at pH 7.4 and pH 4.5, respectively. The introduction of CNWs into the MET–ALG system not only prolonged the drug release, but also increased the mucoadhesive properties by about 1.3 times. Thus, novel CNW–ALG hydrogels are promising carriers for pH sensitive drug delivery carriers.

## 1. Introduction

The rational treatment of vaginal infections requires an effective concentration of antimicrobial agents at the site of infection to prevent disease recurrence. Therefore, topical administration is most commonly recommended due to high local bioavailability, reduced drug dosage and side effects, and self-administration [1]. Nevertheless, topical vaginal drug administration is affected by various physiological factors. First, the vaginal walls are lined with stratified squamous epithelium containing many folds (rugae), increasing the surface absorption area. However, the intra-abdominal pressure collapses the folds, as does the sinuosity of the vaginal canal, which makes it difficult to adequately distribute conventional vaginal dosage forms, such as vaginal gels [2]. Second, cervicovaginal mucus as a physical barrier can affect the penetration, distribution, and residence time of active pharmaceutical ingredients [3]. Therefore, traditional vaginal dosage forms are often characterized by inconsistent distribution, short vaginal residence time, and discomfort, which significantly reduces patient compliance [1]. Appropriately designed delivery systems can improve the vaginal distribution of the drug and provide controlled sustained release [4,5]. For example, containing nanoparticles, bioadhesive vaginal structured microgels based on natural polymers have rheological and mucoadhesive properties [6], which improves the distribution of the drug over the vagina surface and increases the drug residence time [7]. Such formulations provide a controlled and sustained drug release profile [8,9,10].

Chitin nanowhiskers (CNWs), obtained by partial deacetylation of α-chitin, have a unique nanoscale structure and excellent mechanical and adsorption properties [11,12]. CNWs have a cationic nature due to the presence of amino groups on their surfaces; they retain the useful characteristics of both chitin and chitosan, showing excellent biocompatibility, biodegradability, low toxicity, and immunogenicity, as well as antibacterial/antifungal activity [13,14]. CNWs have been used for the fabrication of various biopolymeric scaffolds (such as films [15], electrospun mats [16], and cryogels [17]) for tissue engineering applications. Active functional groups (OH and NH_2_ groups) and a large surface area facilitate the chemical modification of CNWs to obtain novel nanoscale materials [18]. Due to their positive charges, CNWs have mucoadhesive properties and can interact with anionic polysaccharides to form various polymeric structures, such as polyelectrolyte complexes (PECs) and cross-linked polymeric systems [19,20,21]. Therefore, CNWs are widely used as bioadhesive polymer drug delivery platforms with the controlled release of active pharmaceutical ingredients [22,23,24,25]. For example, Lin et al. [26] developed microcapsules based on calcium-cross-linked sodium alginate (ALG) and various polysaccharide crystals such as CNWs, nanocellulose, and nanostarch; the included polymeric nanocrystals enhanced the mechanical properties of the obtained systems due to both high crystallinity and hydrogen bonds with the ALG matrix. The resulting microparticles had pH-sensitive swelling and drug release; due to the modified release, 90% of theophylline was gradually released at pH 7.4 within 12 h. Petrova et al. [19] used CNWs to improve the mechanical and biopharmaceutical properties of ALG hydrogels. It was shown that increasing the CNW content in the hydrogel enhanced the yield stress, maximum Newtonian viscosity, and relaxation time, and also prolonged the tetracycline release within 24 h.

The aim of this work was to develop a hydrogel, based on polyelectrolyte complexes of CNWs and ALG, capable of increasing the efficiency of drug delivery due to prolonged pH-sensitive release of the drug and high mucoadhesion. We chose ALG as the anionic biopolymer; due to its properties of biocompatibility, low toxicity, biodegradability, and chelating ability, ALG is widely used to develop mucoadhesive drug delivery systems, including antimicrobial and antifungal drugs with modified release [27,28]. In addition, ALG has excellent pH sensitivity due to the presence of a carboxylic acid group on its structures [29]. Metronidazole (MET) was used as a model drug to study the nature and rate of drug release from hydrogels based on PECs of CNWs and ALG with dependence on pH. MET is an antiprotozoal and antimicrobial (anaerobic microorganisms) agent; moreover, MET in gel form is the drug of choice for the treatment of bacterial vaginosis [10,30,31,32]. The inclusion of MET in micro or nanoscale delivery systems (e.g., mucoadhesive polymer particles, micro- and nanogels) is a promising strategy to improve its pharmaceutical properties (increased bioavailability, targeted delivery, prolonged action, longer drug residence time in the infectious inflammation site, and reduced degree and frequency of side effects), as well as enhancing the pharmacological activity [33,34,35,36]. For example, chitosan-ALG complexes have been successfully used to develop vaginal inserts or tablets [37,38] and to produce microspheres for vaginal delivery of MET [39]. However, to the best of our knowledge, there are no data on the use of CNW–ALG hydrogels for vaginal delivery of MET.

## 2. Materials and Methods

### 2.1. Materials

We used sodium alginate (Qingdao Bright Moon Seaweed Group Co., LTD, Qingdao, China) with a molecular weight (MW) of 130,000. CNWs were obtained by partial deacetylation of α-chitin, as previously reported [18]. The obtained CNWs had a diameter of 6–15 nm and a length of 100–500 nm, and a degree of deacetylation (DDA) of 0.40 ± 0.03 [18].

The metronidazole, lactic acid, mucin (type II), phosphate-buffered saline (PBS), periodic acid, basic fuchsin, and sodium pyrosulfite were from Sigma-Aldrich (St. Louis, MI, USA); the 1 M hydrochloric acid solution and glacial acetic acid were from Acros Organics (Waltham, MA, USA).

### 2.2. Preparation of CNW–ALG Hydrogels

To prepare CNW–ALG, we used a 0.2% aqueous dispersion of CNWs obtained by magnetic stirring in deionized water for 5 days, followed by sonication; then, 0.5 mL of the CNW dispersion (1 mg of CNWs) was added to 20 mL of deionized water and sonicated at 20 W for 20 min (Bandelin Sonopuls mini 20, Bandelin Electronics, Berlin, Germany). To the resulting CNW dispersion, 5 mL of ALG solution (1 mg/5 mL or 2 mg/5 mL) and acetic acid solution were added for the protonation of amino groups (0.1% and 2% acetic acid solution to pH 5.5 and 3.5, respectively). The reaction mixture was stirred for 1 h, frozen for 24 h, and then freeze dried using a Freeze Dryer 10 N (Fanbolun Ltd., Guangzhou, China).

By varying the reaction conditions (component ratio and pH), we obtained CNW–ALG hydrogels with different physicochemical characteristics. In summary, the synthetic conditions were as follows:

(i) CNW:ALG weight ratio 1:2 at pH 5.5 (CNW–ALG_1_);

(ii) CNW:ALG weight ratio 1:2 at pH 3.5 (CNW–ALG_2_);

(iii) CNW:ALG weight ratio 1:1 at pH 3.5 (CNW–ALG_3_).

Finally, the freeze-dried samples (CNW–ALG_1_, CNW–ALG_2_, and CNW–ALG_3_) were washed with deionized water and separated by MPW-308R centrifuge (MPW Med. Instruments, Warszawa, Poland) at 4500 rpm to remove unbound ALG (CNW–ALG_1w_, CNW–ALG_2w_, and CNW–ALG_3w_ microgels, respectively). Thus, the washed CNW–ALG_1w_, CNW–ALG_2w_, and CNW–ALG_3w_ microgels do not contain unbound ALG.

### 2.3. MET Loading into the CNW–ALG

To prepare MET-loaded CNW–ALG, 80 µL of 1 mg/mL MET aqueous solution was injected into the reaction mixture of CNW and ALG in various ratios and at different pH (obtained according to Section 2.2), before the addition of acetic acid (MET–CNW–ALG_1_, MET–CNW–ALG_2_, and MET–CNW–ALG_3_ samples). The obtained mixtures were stirred for 1 h, frozen for 24 h, and then freeze dried. In addition, the system of MET with ALG was obtained according to the same technique, but without CNWs (MET–ALG_1_), and was prepared as a control (release kinetics and mucoadhesion). The loading efficiency of MET (LE, %) and the MET content (µg/mg) were calculated using the Equations (1) and (2), respectively:

(1) $$\mathrm{LE}\;(\%)=\frac{m\left(\mathrm{MET}\right)\times 100}{m\left(\mathrm{CNW}\right)+m\left(\mathrm{ALG}\right)}$$

(2) $$\mathrm{MET}\;\mathrm{content}\;(\mu g/\mathrm{mg})=\frac{m\left(\mathrm{MET}\right)\times 1000}{m\left(\mathrm{CNW}\right)+m\left(\mathrm{ALG}\right)+m\left(\mathrm{MET}\right)}$$

### 2.4. General Methods of CNW–ALG and MET–CNW–ALG Characterization

Elemental analysis was performed on a Vario EL (Elementar, Hanau, Germany) CHN analyzer. The molar ratio between monomeric units of ALG and CNWs was determined through elemental analysis using the following equation:

(3) $$\frac{1}{x}\left(\frac{\omega C}{\omega N}\left(\mathrm{CNW}–\mathrm{ALG}\right)-\frac{\omega C}{\omega N}\left(\mathrm{CNW}\right)\right)\frac{MW\left(N\right)}{MW\left(C\right)}$$

where *x* is the number of C atoms in the monomeric units of ALG (*x* = 6), *ω* is the mass fraction of the corresponding element, and MW is the molecular weight.

X-ray diffraction was performed on a DRON-3M instrument (Burevestnik, St. Petersburg, Russia) using Ni-filtered Cu Kα radiation (λ = 1.5418 Å).

The apparent hydrodynamic diameter (Dh) and ζ-potential were estimated by dynamic and electrophotoretic light scattering using a Photocor Compact-Z instrument (Photocor, Moscow, Russia) with an He-Ne laser and a wavelength of 659 nm at a detection angle of 90°.

The morphology of the particles was studied by scanning electron microscopy (SEM), which was performed on a Tescan Mira 3 scanning electron microscope (Tescan, Brno, Czech Republic). The samples were placed on a double-sided carbon tape and dried in a vacuum oven for 24 h before SEM studies. Images were acquired in the secondary electron mode at an accelerating voltage of 20 kV and an operating electric current of 550 pA; the distance between the sample and the detector was 6 mm (magnification up to 165,000×).

### 2.5. MET Release Kinetics

The drug release conditions were chosen considering the physiological characteristics of the vagina environment (acidic pH, due to the presence of lactic acid, and body temperature) [40]. A 1 mg sample of MET–CNW–ALG was dispersed in lactic acid solution (2.5 mL, pH 4.5) and in PBS solution (2.5 mL, pH 7.4) and then incubated at 37 ± 0.5 °C. At regular intervals, 2.5 mL of medium was ultracentrifuged (MPW-308R centrifuge, MPW Med. Instruments, Warszawa, Poland) at 4500 rpm, using a 10,000 MWCO Vivaspin^®^ Turbo 4 centrifugal concentrator; then, the same volume of fresh medium was added. The amount of released MET was determined spectrophotometrically (UV-1700 PharmaSpec spectrophotometer, Shimadzu, Kyoto, Japan) in the supernatant at 320 nm with a calibration curve.

### 2.6. Mucoadhesion of MET–CNW–ALG and MET–ALG

The mucoadhesive properties were studied by mucin adsorption using the two-step periodic acid/Schiff colorimetric method [41,42]. The periodic acid was prepared as follows: 10 μL of 50% periodic acid was added to 7 mL of 7% acetic acid. The Schiff reagent was prepared as follows: 100 mL of 1% aqueous basic fuchsin was added to 20 mL of 1 M HCl; then, the resulting mixture was decolorized twice with 300 mg of activated charcoal for 5 min. Sodium pyrosulfite (0.1 g per 6 mL of Schiff reagent) was added directly before use and the resulting solution was incubated at 37 °C until it became colorless or pale yellow (about 90–100 min).

The calibration curve was obtained as follows: 200 μL of freshly prepared periodic acid was added to 2 mL of standard solutions of mucin (0.02–0.08 mg/mL). The resulting solutions were incubated at 37 °C for 120 min to complete the periodate oxidation; then, colorless Schiff reagent (200 μL) was added and left for 30 min at room temperature (the solution turned pink). The absorbance of the standard solutions was measured at 565 nm using a UV–Vis spectrophotometer (UV-1700 PharmaSpec, Shimadzu, Kyoto, Japan).

Mucin solution (0.5 mg/mL; 1 mL) was added to the MET–CNW–ALG_1_ and MET–ALG_1_ (0.5 mg/mL; 10 mL) with magnetic stirring at 500 rpm, and the mixture was incubated at 37 °C for 60 min. The resulting mixture was centrifuged at 4500 rpm for 60 min, and the supernatant was used to measure the free mucin concentration, using the calibration curve. A solution containing all the components of the analyzed solution, except for the analyte, was used as a reference solution. The mucoadhesiveness (the mucin binding efficiency) was calculated from the following equation:

(4) $$\mathrm{Mucoadhesiveness}\;(\%)=\frac{\left(C_{o}-C_{s}\right)\times 100}{C_{o}}$$

where *C_o_* is the initial mucin concentration and *C_s_* is the mucin concentration in the supernatant.

## 3. Results and Discussion

### 3.1. Preparation and Characterization of CNW–ALG and MET–CNW–ALG Hydrogels

**Figure 1:**
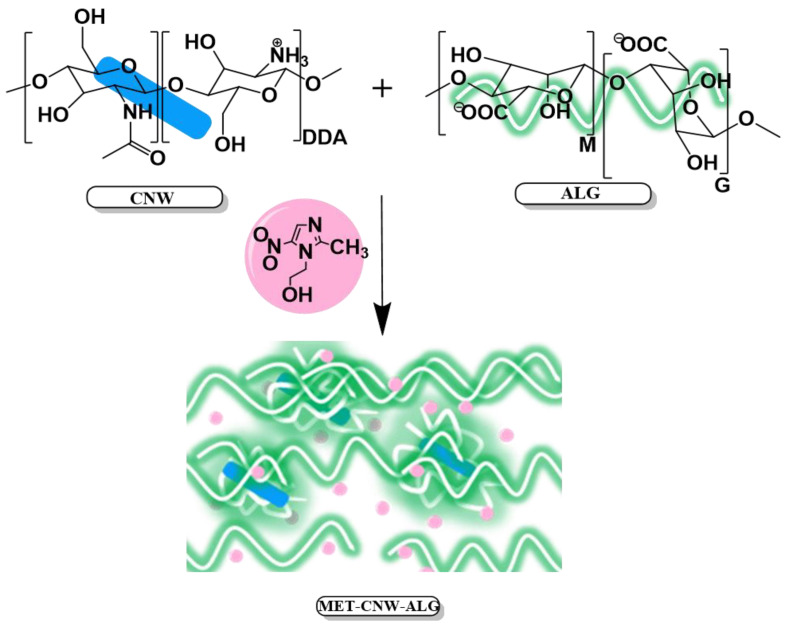
Formation of MET–CNW–ALG hydrogels.

PEC between CNWs and ALG is formed due to various chemical and physical interactions between these polymers (Figure 1). ALG is a negatively charged polysaccharide consisting of 1⟶4 linked sodium α-L-guluronate (G) and β-D-mannuronate (M), which can be arranged in heteropolymeric (MG) and homopolymeric (G and M) blocks; the ratio between and G monomeric units varied widely [43,44]. We assume that CNW–ALG particles are formed through electrostatic interactions between CNWs and ALG (through the interpolymer interaction between the protonated amino groups of CNWs and the carboxyl groups of ALG); then, physical hydrogels are formed by both the molecular entanglement of ALG chains and the formation of additional hydrogen bonds (Figure 1).

The composition of the CNW–ALG hydrogels and separated microgels was determined using an elemental analysis data (Table 1). It was shown that hydrogels (CNW–ALG_1_, CNW–ALG_2_, and CNW–ALG_3_) are enriched with ALG compared to washed microgels, which are CNW and ALG PECs (CNW–ALG_1w_, CNW–ALG_2w_, and CNW–ALG_3w_). The samples CNW–ALG_1_ and CNW–ALG_2_ (component ratio of 1:2) contain a two-fold ALG amount compared to sample CNW–ALG_3_ (component ratio of 1:1), regardless of the pH of the reaction mixture.

At the same time, the PEC compositions depended on the pH of the reaction mixture (Table 1). Thus, when the pH changed from 3.5 to 5.5, the amount of bound ALG in PEC increased almost three-fold (CNW–ALG_2w_ and CNW–ALG_1w_ microgels, respectively). In general, the ALG:CNW molar ratio of 0.37 for CNW–ALG_1w_ microgel (pH 5.5) indicated that ALG was bound to nearly all amino groups of CNWs (DDA = 0.40).

**Figure 2:**
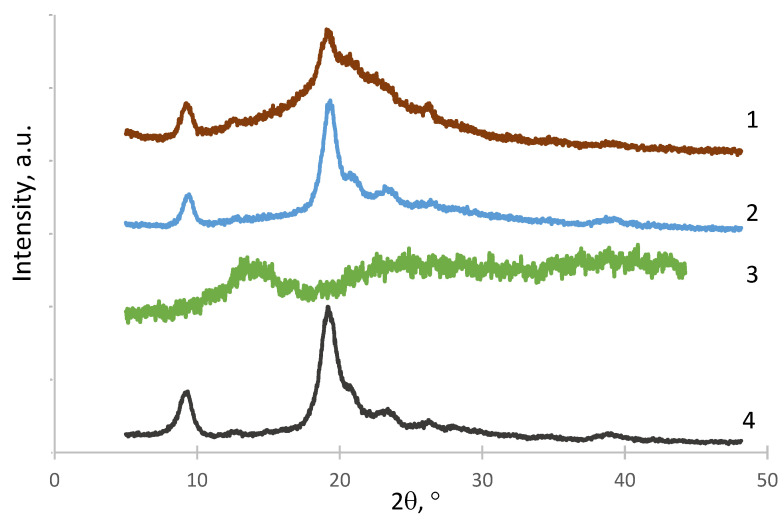
X-ray diffractograms of CNW–ALG_1_ (1), CNW–ALG_1__w_ (2), ALG (3), and CNWs (4).

The structure of the obtained polymer complexes was studied by X-ray diffraction (Figure 2). The X-ray diffractogram of the CNW–ALG_1_ sample (Figure 2, curve 1) had a reflex at the 2Ɵ region of 20–25° and a reflex at the 2Ɵ = 9° due to the influence of both the CNWs (Figure 2, curve 4) and the ALG (Figure 2, curve 3) structures. The structure of the CNW–ALG_1w_ sample (Figure 2, curve 2) was similar to that of the original CNWs showing the disappearance of the ALG structure after the washing of the CNW–ALG_1_ sample.

The prepared polyelectrolyte systems were characterized by their Dh and ζ-potential (Table 2). As shown in Table 2, the initial CNWs had a positive charge and a hydrodynamic size of 300 nm; in addition, there was a small fraction with a Dh of about 50 nm (further, it was characteristic of all obtained samples). The microparticles in the hydrogel samples had a negative surface charge (−39 to −25 mV) and hydrodynamic size decreasing in the series CNW–ALG_1_ > CNW–ALG_2_ > CNW–ALG_3_; thus, larger microparticles (CNW–ALG_1_) were formed at reaction pH 5.5 and with a CNW:ALG ratio of 1:2. The washed microgel particles also had a negative charge (−27 to −22 mV) and Dh with the same dependence pattern on the reaction conditions in the series CNW–ALG_1w_ > CNW–ALG_2w_ > CNW–ALG_3w_. Using the CNW–ALG_1_ sample as an example, it was shown that ALG-modified CNW dispersions were stable overnight at human body temperature (Table 2).

Furthermore, the systems loaded with MET at pH 3.5 (MET–CNW–ALG_2_ and MET–CNW–ALG_3_) almost did not change their Dh compared to the unloaded particles (CNW–ALG_2_ and CNW–ALG_3_, respectively), whereas the Dh of MET-containing complexes formed at pH 5.5 increased approximately 1.7-fold versus MET-free microparticles (MET–CNW–ALG_1_ and CNW–ALG_1_, respectively).

**Figure 3:**
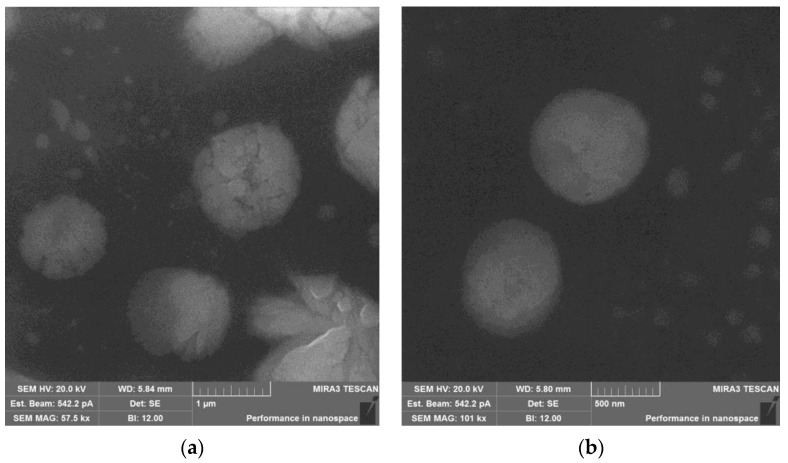
SEM image of CNW–ALG_1_ (**a**) and MET–CNW–ALG_1_ (**b**).

The obtained SEM images (Figure 3) of the samples correlated with the dynamic light scattering data (Table 2). The CNW–ALG_1_ particles had sizes of 0.7–1.2 µm and friable surface, whereas the MET-loaded particles (MET–CNW–ALG_1_) had a denser morphology and their size (0.8–1.3 µm) decreased during drying approximately 1.7 times, as compared to Dh (Table 2). A smaller fraction was also present in both samples, probably consisting of the initial CNWs with a small amount of ALG.

### 3.2. MET Release Kinetics from the MET–CNW–ALG

The resulting polymeric complexes had a solid core (CNWs) and an amorphous shell (ALG), and this structure is usually capable of modifying the release profile of hydrophilic drugs [26]. The different swelling ability depending on the pH of the ALG amorphous shell allows the prediction of pH-sensitive drug release. These systems are suitable drug carriers with pH-dependent modified release (the contraction of the particle at a decreasing pH promotes the release of drug molecules) [45].

**Figure 4:**
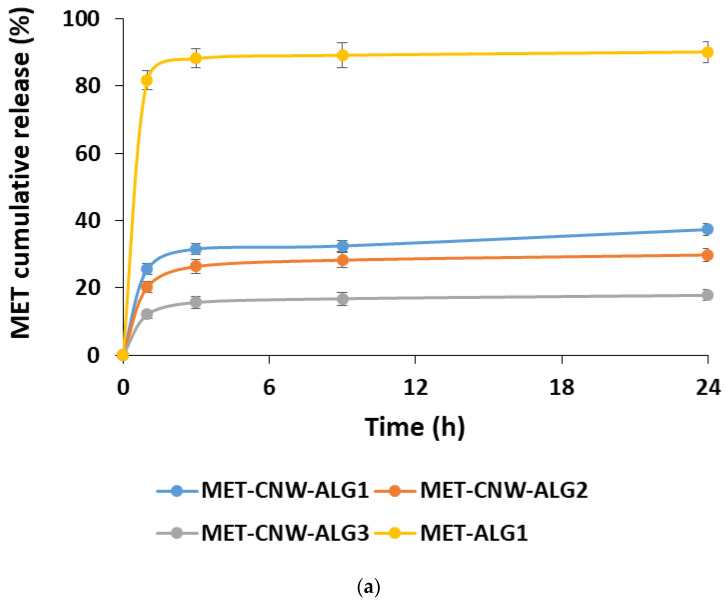

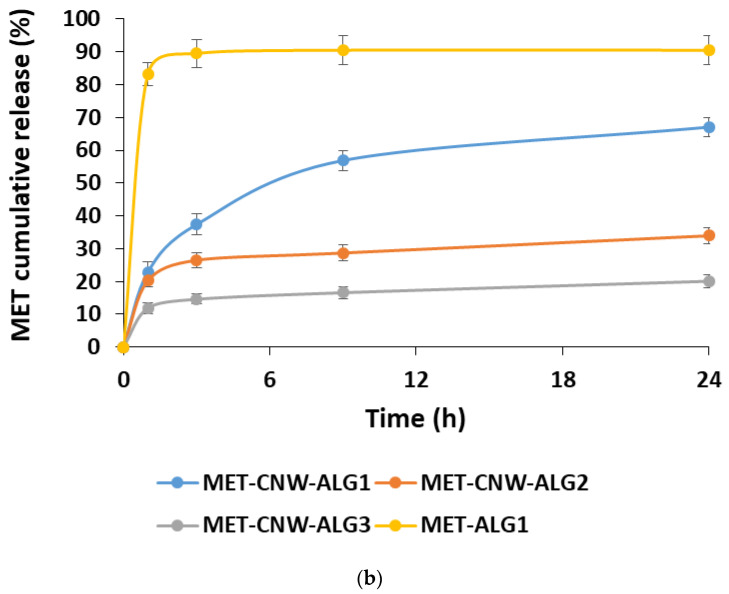
MET release kinetics at 37 °C from the MET–CNW–ALG in PBS, pH 7.4 (**a**) and in lactic acid solution, pH 4.5 (**b**). Data are presented as mean ± standard deviation (*n* = 3).

The kinetics of the MET release was studied using both PBS (pH 7.4) and a lactic acid solution (pH 4.5), which simulates the conditions of vaginal delivery. In this case, the effect of the formation conditions on the MET release rate was studied (Figure 4).

The MET release depends on both the structure of the hydrogels and the pH of the release medium. Using the MET–CNW–ALG_1_ and MET–ALG_1_ samples, it was shown that the presence of CNWs significantly prolonged the release of MET compared to the MET–ALG_1_ system. In addition, conditions such as the lower pH of the particle-forming medium and a lower ALG content had a prolonged effect.

The pH of the release medium had almost no effect on the release kinetics of the MET from particles formed at pH 3.5 (MET–CNW–ALG_2_ and MET–CNW–ALG_3_); approximately 15–30% of the substance was released over 24 h at both pH 7.4 and pH 4.5. In contrast, the systems obtained at pH 5.5 (MET–CNW–ALG_1_) had a pH-sensitive release profile; after 24 h, a two-fold higher MET amount was released into the lactic acid medium with pH 4.5 (simulating the vaginal environment) than into the PBS with pH 7.4. This pH-dependent release is probably due to the fact that large particles with an amorphous ALG shell contract in an acidic medium and more easily release MET, making these polymeric hydrogels suitable for use at an acidified pH, for instance, as vaginal delivery systems.

### 3.3. Mucoadhesion of MET–CNW–ALG and MET–ALG

**Figure 5:**
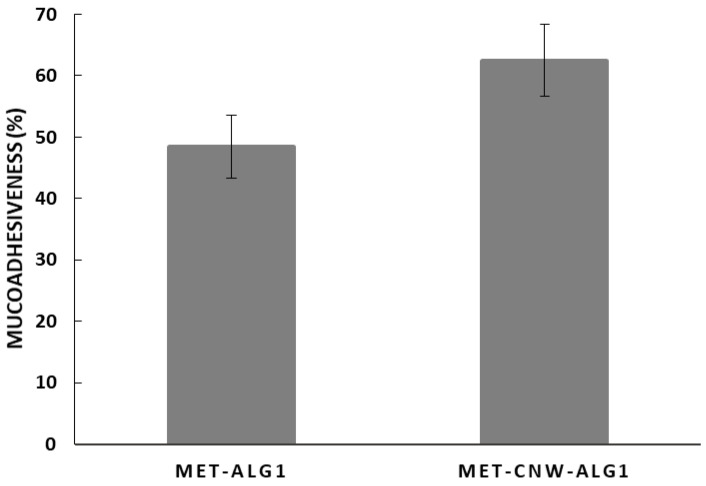
Mucoadhesiveness of MET–CNW–ALG_1_ and MET–ALG_1_. Each column represents the average of triplicate measurements ± standard deviation.

Mucoadhesive drug delivery systems adhere to the vaginal mucosa, thereby increasing drug residence time and bioavailability, while also providing controlled drug release and prolonging the therapeutic effect [9]. Both CNWs and ALG have an intermolecular interaction with various functional groups of mucin via hydrogen bonds and polymer entanglement, as well as electrostatic bindings and hydrophobic interaction [46,47]. We studied the mucoadhesion of the sample MET–CNW–ALG_1_, since it proved to be the most promising delivery system in terms of release profile; in addition, we compared the mucoadhesion of the MET–CNW–ALG_1_ system with the CNW-free system. The mucoadhesive properties were evaluated through the ability of polymeric systems to bind mucin in aqueous solution. The adsorbed amount of mucin was measured through the change in free mucin concentration in the supernatant according to the Equation (4). It was shown (Figure 5) that both MET–CNW–ALG and MET–ALG effectively bound mucin (mucoadhesiveness was approximately 60 and 50%, respectively); however, the use of CNWs increased the mucoadhesion ability.

Thus, due to the ability to bind to mucin, the MET–CNW–ALG hydrogels can prevent the rapid excretion of the active pharmaceutical ingredient through vaginal secretions. This indicates their prospects in the antimicrobial therapy of bacterial vaginosis.

## 4. Conclusions

In this work, we developed polysaccharide hydrogels for potential application as pH-sensitive prolonged-release vaginal delivery systems. We first prepared hydrogels based on CNWs and ALG using different molar ratios (1:1 and 1:2) and with the varying pH of the reactant mixtures (3.5 and 5.5); then, we loaded them with the model drug, MET. With an increase in both the pH of particle formation and the amount of ALG in the system, the Dh of the CNW–ALG microparticles in the hydrogel composition increased, varying from 500 to 1100 nm. The prepared particles changed their Dh depending on their pH; thus, when the pH decreased from 9.3 to 2.4, the Dh of the particles decreased approximately two-fold. This particle property optimally provided pH-sensitive MET release from the obtained friable amorphous systems in an acidic environment simulating vaginal pH. CNW acts as a solid core in the CNW–ALG microparticles in the hydrogel, prolonging drug release compared to the CNW-free systems. At a target vaginal pH of 4.5, the MET release was about 65–67% for 24 h. The developed vaginal delivery system had favorable mucoadhesive properties, thereby increasing its residence time in the vagina; in addition, the microgel structure can improve the biodistribution of this polymeric system over the vaginal mucosa, providing controlled and prolonged release and, thus, improving the biopharmaceutical properties of the drug included in the polymeric carrier. We suggest that CNW–ALG hydrogels can be considered as promising drug delivery carriers for topical administration, including the treatment of vaginal infections.

## Figures and Tables

**Table 1:** Preparation conditions and composition of CNW–ALG PECs.

Sample	pH and CNW:ALG Mass Ratio	Elemental Analysis (%)		ALG:CNW Ratio (mol/mol Monomeric Units)
		C	N	
CNW	-	44.20	6.50	-
ALG	-	29.30	-	
CNW–ALG_1_	pH = 5.5	33.98	1.81	2.30
CNW–ALG_1w_	1:2	41.30	4.74	0.37
CNW–ALG_2_	pH = 3.5	35.70	1.90	2.30
CNW–ALG_2w_	1:2	40.20	5.32	0.14
CNW–ALG_3_	pH = 3.5	34.40	2.70	1.10
CNW–ALG_3w_	1:1	40.71	5.49	0.12

**Table 2:** Physicochemical parameters of CNW–ALG and MET–CNW–ALG. Data represent mean ± standard deviation (*n* = 5).

Sample	Dispersion pH	Dh (nm)	ζ-Potential (mV)	MET Content (μg/mg)	LE (%)
CNW	6.3	50 ± 30, 300 ± 50	+20 ± 0.5	-	
CNW–ALG_1_	6.3	85 ± 8, 1104 ± 306	−27.5 ± 1.1	-	
		110 ± 24, 1066 ± 304 *	−30.1 ± 2.0 *		
CNW–ALG_1w_	9.3	57 ± 12, 302 ± 72	−21.8 ± 0.1	-	
	6.3	77 ± 13, 233 ± 54	−22.4 ± 0.1		
	2.4	20 ± 2, 142 ± 25	−5.6 ± 0.2		
MET–CNW–ALG_1_	6.3	99 ± 10, 1738 ± 356	−42.7 ± 2.7	26	2.7
		102 ± 12, 1618 ± 412 *	−40.7 ± 1.9 *		
CNW–ALG_2_	6.3	57 ± 11, 604 ± 119	−38.9 ± 0.2	-	
CNW–ALG_2w_	6.3	216 ± 68	−26.5 ± 0.3	-	
MET–CNW–ALG_2_	6.3	75 ± 22, 648 ± 106	−29.7 ± 1.5	26	2.7
CNW–ALG_3_	6.3	63 ± 191, 474 ± 98	−25.4 ± 0.3	-	
CNW–ALG_3w_	6.3	190 ± 54	−23.1 ± 0.8	-	
MET–CNW–ALG_3_	6.3	40 ± 4, 520 ± 102	−15.6 ± 1.3	38	4.0

* Parameters after exposure at 37 °C for 24 h.

## Data Availability

Data are contained within the article.
